# HIV Prevalence and Incidence among Sexually Active Females in Two Districts of South Africa to Determine Microbicide Trial Feasibility

**DOI:** 10.1371/journal.pone.0021528

**Published:** 2011-08-10

**Authors:** Annaléne Nel, Cheryl Louw, Elizabeth Hellstrom, Sarah L. Braunstein, Ina Treadwell, Melanie Marais, Martie de Villiers, Jannie Hugo, Inge Paschke, Chrisna Andersen, Janneke van de Wijgert

**Affiliations:** 1 International Partnership for Microbicides, Silver Spring, Maryland, United States of America; 2 Madibeng Centre for Research, Brits, South Africa; 3 Be Part Yoluntu Centre, Mbekweni and Paarl, South Africa; 4 New York City Department of Health and Mental Hygiene, New York City, New York, United States of America; 5 Academic Medical Center of the University of Amsterdam and Amsterdam Institute for Global Health and Development, Amsterdam, The Netherlands; Boston University, United States of America

## Abstract

**Background:**

The suitability of populations of sexually active women in Madibeng (North-West Province) and Mbekweni (Western Cape), South Africa, for a Phase III vaginal microbicide trial was evaluated.

**Methods:**

Sexually active women 18–35 years not known to be HIV-positive or pregnant were tested cross-sectionally to determine HIV and pregnancy prevalence (798 in Madibeng and 800 in Mbekweni). Out of these, 299 non-pregnant, HIV-negative women were subsequently enrolled at each clinical research center in a 12-month cohort study with quarterly study visits.

**Results:**

HIV prevalence was 24% in Madibeng and 22% in Mbekweni. HIV incidence rates based on seroconversions over 12 months were 6.0/100 person-years (PY) (95% CI 3.0, 9.0) in Madibeng and 4.5/100 PY (95% CI 1.8, 7.1) in Mbekweni and those estimated by cross-sectional BED testing were 7.1/100 PY (95% CI 2.8, 11.3) in Madibeng and 5.8/100 PY (95% CI 2.0, 9.6) in Mbekweni. The 12-month pregnancy incidence rates were 4.8/100 PY (95% CI 2.2, 7.5) in Madibeng and 7.0/100 PY (95% CI 3.7, 10.3) in Mbekweni; rates decreased over time in both districts. Genital symptoms were reported very frequently, with an incidence of 46.8/100 PY (95% CI 38.5, 55.2) in Madibeng and 21.5/100 PY (95% CI 15.8, 27.3) in Mbekweni. Almost all (>99%) participants said that they would be willing to participate in a microbicide trial.

**Conclusion:**

These populations might be suitable for Phase III microbicide trials provided that HIV incidence rates over time remain sufficiently high to support endpoint-driven trials.

## Introduction

At the end of 2009, about 7,000 new HIV infections occurred each day [1]. New HIV prevention tools, especially those that women can use, are therefore desperately needed. Microbicides are being developed for topical application inside the vagina or rectum to prevent infection with HIV and possibly other sexually transmitted infections (STIs) [2]. Microbicide research has been ongoing for about 20 years. Proof-of-concept for vaginal microbicides was obtained in 2010, when the CAPRISA 004 trial showed a 39% reduction in HIV incidence after 30 months of tenofovir gel use compared to placebo gel use [3]. Phase III clinical trials of candidate microbicides are often conducted in sub-Saharan Africa because this is where 70% of the new HIV infections occur [1]. In preparation for such trials, estimates of HIV incidence in target populations are needed to determine adequate sample size and statistical power for demonstrating safety and efficacy [4].

South Africa has hosted, and is hosting, several microbicide and other HIV prevention intervention trials. While HIV prevalence data are available for many districts of South Africa, they are not available for all potential microbicide trial populations. HIV incidence data are hard to find [5]. Madibeng and Mbekweni are two districts of South Africa that had not yet participated in HIV prevention intervention trials when the studies described in this paper were conducted, and HIV prevalence and incidence where not yet known. Madibeng is a rural district municipality in North-West Province supported by mining (chrome, granite, and platinum), manufacturing (automotive, metal, and fuel), and agriculture. Mbekweni is a small urban township close to Paarl in the Western Cape; many of its residents are employed in the deciduous farming and wine-making industry. In both districts, the community is somewhat migratory because of unstable employment.

## Methods

### Study design and populations

**Figure 1:**
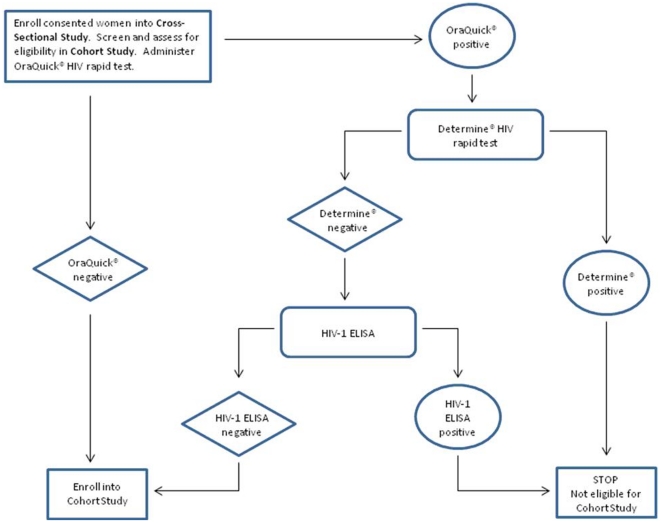
HIV testing algorithm. Approximately 800 women at each CRC were tested for HIV infection at screening as indicated. Those confirmed as seronegative and who met the entry criteria (299 at each CRC) were enrolled into the prospective cohort study and retested at 3, 6, 9, and 12 months after enrollment using the same algorithm. Participants who became HIV-positive while on study were referred to available sources of psychosocial and medical care and support. HIV-positive participants could continue on study for scheduled examinations per protocol with the exception of any further HIV testing and genital assessment, unless clinically indicated.

HIV prevalence and incidence in two districts of South Africa, Madibeng and Mbekweni, were estimated in cross-sectional studies (targeting 800 women) followed by prospective cohort studies (targeting 300 women) to determine the suitability of the populations for participation in Phase III microbicide trials. Women were recruited from local family planning clinics in Madibeng and from family planning clinics, community events, and door-to-door visits in Mbekweni. The clinical research centers (CRCs) used recruitment strategies that they also plan to use in future Phase III microbicide trials and these were CRC-specific. However, the same study procedures were followed at each CRC from the moment women visited the CRC to be screened for study participation. Women were eligible for the cross-sectional studies if they were 18–35 years, not HIV-positive or pregnant by self-report, not breastfeeding, and sexually active (defined as at least one penetrative vaginal coital act per month for the previous three months). At cross-sectional study visits, eligible women were tested for HIV antibodies using a rapid testing algorithm (Figure 1) and for pregnancy. Women who tested positive for HIV antibodies were also tested by BED capture enzyme immunoassay (BED) to determine the proportion of recent infections [6].

Women who tested HIV- and pregnancy-negative in the cross-sectional cohort studies, still met the entry criteria described above, and met additional entry criteria for the cohort studies were subsequently offered enrollment into the cohort studies. These additional entry criteria included using a reliable WHO-approved contraceptive method [7], not injecting non-therapeutic drugs, not participating in other studies, not suffering from specified chronic diseases or allergies, refraining from anal sex and planning to stay in the study area for the duration of the study. Follow-up visits occurred after 3, 6, 9 and 12 months. Screening continued until 800 women were enrolled in the cross-sectional studies and 300 HIV-negative women were enrolled in the cohort studies at each CRC.

All women in the cross-sectional and cohort studies (at all study visits) were interviewed regarding demographics, sexual behavior, and medical history; and received HIV risk reduction and contraceptive counseling, condoms, and syndromic management of sexually transmitted infections (STI) free of charge [8]. Confirmed HIV-positive women were referred for HIV care, and pregnant women were referred for antenatal care, but HIV-positive and pregnant women were retained in the study. The study was approved by Pharma-Ethics in South Africa. Written informed consent was obtained from all study participants.

### Laboratory testing

Oral swabs from each participant were tested using OraQuick ADVANCE Rapid HIV-1/2 Antibody Test (OraSure Technologies, Inc., Bethlehem, PA, USA). Blood samples from women with positive OraQuick results were tested by Determine HIV-1/2 rapid test (Inverness Medical Professional Diagnostics, Princeton, NJ, USA), and by enzyme-linked immunosorbant assay (ELISA) if a tiebreaker was needed. Blood samples from women who were confirmed HIV-positive were also tested by BED assay (Calypte Biomedical Coorporation, Portland, OR, USA) according to the manufacturer's instructions. A specimen with a final normalized optical density value of less than or equal to 0.8 was considered to be from a patient who was infected less than 155 days ago [6].

### Data Analysis and Statistics

#### Sample size calculations

In the cross-sectional studies, a sample size of 800 women would allow for a precision level of the HIV incidence estimate of 4.0/100 PY±3.5, 5.0/100 PY±3.7, and 6.0/100 PY±3.9 assuming an HIV prevalence of 25% and using the McWalter and Welte formula described below [9], [10]. In the cohort studies, accumulation of 270 personyears (PY) of follow up (300 women for one year minus 10% PY lost to follow-up) would generate the following 95% confidence intervals around the observed HIV incidence rates: 11 seroconversions, 4.1 (95% CI 1.7, 6.5); 14 seroconversions, 5.2 (95% CI 2.5, 7.9); 17 seroconversions, 6.3 (95% CI 3.3, 9.3).

#### Statistical analysis

Data were double entered and analyzed using SAS version 9.2 (SAS Institute, Cary, NC). Descriptive statistics were used to summarize baseline demographic, behavioral and clinical characteristics. Categorical variables are expressed as percentages, and continuous data as medians with inter-quartile ranges.

HIV, pregnancy, and STI symptom incidence rates in the cohort studies were calculated based on a Poisson distribution with PY at risk in the denominator. A person's time at risk began at the enrollment visit and ended at the last study visit attended (usually the Month 12 visit) or when HIV infection or pregnancy occurred. HIV infection and pregnancy were assumed to have occurred at the mid-point between the last available negative test and first positive test. A woman who reached an HIV endpoint was no longer considered at risk for HIV but was still considered at risk for pregnancy, and vice versa. Incident genital symptom cases were defined as participants ever reporting a symptom during follow-up.

HIV incidence rates and 95% confidence intervals based on BED results in the cross-sectional studies were calculated using the formula, and accompanying spreadsheet, provided by McWalter and Welte [9], [10]. Inputs in the formula include the total number of HIV-positive and HIV-negative individuals in the sample, the number of HIV-positive individuals who also tested positive on the BED assay, the BED window period (155 days), and an estimated BED false-recent rate of 5.2% [11]. Incidence estimates are expressed as an incidence rate (number of new HIV infections per 100 PY).

Age-adjusted logistic regression models were used to assess predictors of prevalent HIV infection and pregnancy, with p-values from the Wilcoxon-Mann-Whitney test for continuous variables and the Chi-square and Fisher's exact tests for categorical variables. Age-adjusted Cox proportional hazards regression models were used to assess predictors of HIV seroconversion and incident pregnancy.

## Results

### Disposition

Between April 2007 and March 2008, 798 women were enrolled in the cross-sectional study in Madibeng and 800 in Mbekweni; 299 women at each CRC were subsequently enrolled in the cohort studies. In the cohort studies, total PY of follow-up were 258.4 and 250.8 in Madibeng and Mbekweni, respectively. In Madibeng, 254 of 299 (85%) participants completed all scheduled visits; 17 women withdrew early from the cohort study, 15 were lost to follow-up, 13 missed a scheduled visit, and none died. In Mbekweni, 229 of 299 (77%) participants completed all scheduled visits; 23 women withdrew early from the cohort study, 22 were lost to follow-up, 25 missed a scheduled visit, and none died.

### Demographic Characteristics

In the cross-sectional studies, the median age of study participants at each CRC was 24 years (Table 1). Most participants were black African, single, and had at least some high school education. Over 85% of participants at each CRC had one male sexual partner in the previous 3 months, and less than half (44–45%) used a condom during their last sex act. Twice as many participants in Mbekweni (32%) as Madibeng (16%) had a current sexual partner that they knew was HIV-positive. Anal sex was rarely reported at each CRC (<2%), but oral sex was more common (9–13%). The percentage of women cleansing the vagina before or after sex was higher in Madibeng than Mbekweni (6.5% vs 1.4% and 13.0% vs 0.9%, respectively). At each CRC, demographic and sexual behavior characteristics of cohort study participants at enrollment were similar to cross-sectional participants with one exception: in Madibeng, fewer women in the cohort than in cross-sectional study felt that they were at high risk for HIV (21% vs. 41%).

**Table 1:** Baseline Characteristics of Study Participants.

	Cross-Sectional Studies		Cohort Studies	
	Madibeng	Mbekweni	Madibeng	Mbekweni
Characteristic n (%)	N = 798^1^	N = 800	N = 299^2^	N = 299^3^
Age in years (median)	24	24	23	23
Age in years				
18–20	202 (25.3)	172 (21.5)	95 (31.8)	90 (30.1)
21–25	316 (39.6)	283 (35.4)	120 (40.1)	110 (36.8)
26–30	159 (19.9)	195 (24.4)	53 (17.7)	57 (19.1)
31–35	121 (15.2)	150 (18.8)	31 (10.4)	42 (14.1)
Race				
Black African	744 (93.2)	711 (88.9)	270 (90.3)	292 (97.7)
Other	54 (6.8)	89 (11.1)	29 (9.7)	7 (2.3)
Marital status				
Married/living together	210 (26.3)	274 (34.3)	61 (20.4)	76 (25.4)
Separated/divorced	3 (0.4)	4 (0.5)	0	2 (0.7)
Widowed	0	1 (0.1)	0	0
Single	590 (73.9)	521 (65.1)	238 (79.6)	221 (73.9)
Education				
No school	2 (0.3)	1 (0.1)	1 (0.3)	0
Some/completed primary school	52 (6.5)	58 (7.3)	9 (3.0)	13 (4.3)
Some/completed high school	728 (91.2)	708 (88.5)	281 (94.0)	272 (91.0)
Some/completed tertiary school	16 (2.0)	33 (4.1)	8 (2.7)	14 (4.7)
Male sex partners in last 3 months				
1	685 (85.8)	759 (94.9)	256 (85.6)	287 (96.0)
2	85 (10.7)	38 (4.8)	32 (10.7)	11 (3.7)
3 or more	27 (3.4)	3 (0.4)	11 (3.7)	1 (0.3)
Male sex partners in last 7 days				
0	43 (5.4)	29 (3.6)	25 (8.4)	12 (4.0)
1	725 (91.4)	761 (95.1)	266 (89.6)	287 (96.0)
2 or more	25 (3.2)	10 (1.3)	6 (2.0)	0
Condom used during last sex act	355 (44.5)	348 (43.5)	138 (46.3)	153 (51.2)
Any chance that any current sex partner is HIV+				
Yes	123 (15.9)	246 (32.2)	34 (11.9)	104 (37.3)
No	413 (53.3)	216 (28.3)	175 (61.0)	45 (16.1)
Don't know	239 (30.8)	301 (39.5)	78 (27.2)	130 (46.6)
Willing to participate in microbicide trial	784 (98.6)	798 (99.9)	294 (98.3)	297 (99.7)
Ever had anal sex	15 (1.9)	12 (1.5)	6 (2.0)	4 (1.3)
Ever had oral sex	103 (12.9)	69 (8.6)	40 (13.4)	42 (14.0)
Ever vaginal cleansing before sex^4^	52 (6.5)	11 (1.4)	19 (6.4)	6 (2.0)
Ever vaginal cleansing after sex^4^	103 (13.0)	7 (0.9)	43 (14.4)	5 (1.7)
Self assessment of HIV risk				
No risk	49 (6.2)	3 (0.4)	28 (9.4)	0
Low risk	259 (32.6)	252 (31.7)	139 (46.5)	88 (29.4)
Moderate risk	87 (11.0)	26 (3.3)	37 (12.4)	3 (1.0)
High risk	327 (41.2)	484 (60.9)	64 (21.4)	204 (68.2)
Don't know	72 (9.1)	30 (3.8)	31 (10.4)	4 (1.4)

1 Two women were not eligible and excluded.

2 One woman was found to be less than 18 years of age after enrollment and was subsequently excluded.

3 One woman enrolled twice using a different name; data from her second enrollment were excluded.

4 Included disinfectants/soaps, cotton wool/wad of cloth, and traditional herbs (Mbekweni only).

### HIV prevalence

HIV prevalence was similar in the two districts: 24.1% (95% CI 12.1, 27.1) in Madibeng and 21.8% (95% CI 18.9, 24.7) in Mbekweni. Factors positively associated with prevalent HIV infection at both CRCs were: primary education as highest educational level achieved, inconsistent condom use in the last 7 days, self-assessment of HIV risk as high, suspected positive or unknown HIV serostatus of a current sexual partner, and presence of STI symptoms at baseline (Table 2). Being married or living together was negatively associated with HIV infection. Other risk factors were significantly associated with prevalent HIV infection at one CRC only (Table 2).

**Table 2:** Determinants of Prevalent HIV Infection in the Cross-Sectional Studies^1^.

Determinant	Madibeng (N = 192)		Mbekweni (N = 174)	
	% HIV+	Age-adjusted OR (95% CI)	% HIV+	Age-adjusted OR (95% CI)
Race				
Black African	24.7	2.1 (1.0, 4.7)	24.3	32.5 (4.5, 235.7)^2^^,^ 3
Other (reference)	14.8		1.1	
Marital status:				
Married/living together	24.3	0.6 (0.4, 0.9)^2^	21.5	0.6 (0.4, 0.9)^2^
Single, separated or divorced (reference)	24.0		21.9	
Highest level of education achieved^4^:				
Some/completed primary education	50.0	8.8 (1.1, 73.5)^2^	34.5	11.9 (1.5, 94.7)^2^
Some/completed high school	22.7	3.0 (0.4, 23.0)	21.5	8.0 (1.1, 59.1)^2^
Some/completed tertiary education (reference)	6.3		3.0	
Source of income:				
Woman herself (reference)	27.6		22.9	
Husband/partner	27.1	1.0 (0.6, 1.7)	20.4	0.8 (0.5, 1.2)
Family	18.1	1.1 (0.6, 2.0)	17.6	1.0 (0.7, 1.6)
Other	29.4	1.4 (0.8, 2.4)	40.3	2.9 (1.6, 5.3)^2^
Average monthly income^5^				
0-R500 (reference)	24.2		37.7	
R501-R1000	19.8	0.6 (0.4, 1.1)	29.2	0.7 (0.4, 1.2)
R1001-R2000	32.0	1.2 (0.8, 2.0)	22.1	0.5 (0.3, 0.9)^2^
>R2000	18.3	0.6 (0.3, 1.1)	12.7	0.2 (0.1, 0.5)^2^
Condom use in last 7 days				
Always (reference)	17.2		14.6	
Inconsistent	28.7	1.7 (1.0, 2.8)^2^	26.7	1.9 (1.1, 3.3)^2^
Never	25.0	1.1 (0.6, 1.8)	15.3	0.7 (0.4, 1.4)
Ever had anal sex				
Yes	46.7	3.3 (1.1, 9.9)^2^	16.7	0.6 (0.1, 3.0)
No (reference)	23.6		21.9	
Ever had oral sex				
Yes	21.4	0.9 (0.5, 1.4)	7.3	0.3 (0.1, 0.7)^2^
No (reference)	24.5		23.1	
Self assessment of HIV risk				
No/low risk (reference)	9.4		11.0	
Moderate risk	27.6	3.2 (1.7, 5.9)^2^	19.2	1.8 (0.6, 5.2)
High risk	35.8	5.1 (3.3, 8.1)^2^	26.5	2.7 (1.7, 4.2)^2^
Any chance that any current sex partner is HIV+				
Yes	50.4	5.5 (3.5, 8.8)^2^	26.0	2.2 (1.3, 3.5)^2^
No (reference)	13.2		13.9	
Don't know	28.5	2.3 (1.5, 3.4)^2^	22.9	1.9 (1.2, 3.1)^2^
Reported STI symptom at baseline				
Yes	31.0	1.6 (1.2, 2.3)^2^	41.8	3.1 (1.9, 5.1)^2^
No (reference)	20.7		19.6	

1 Each row represents one bivariable model including age and the predictor of interest.

2 Age-adjusted odds ratio significantly different for predictor vs. reference value (p<0.05);

3 Only 7 women had a race other than black African (they were Cape coloured).

4 Only 8 women in Madibeng and 14 women in Mbekweni had some/completed tertiary education.

5 R = rand; 1 US dollar = 7.4 South African rands.

### HIV incidence

**Figure 2:**
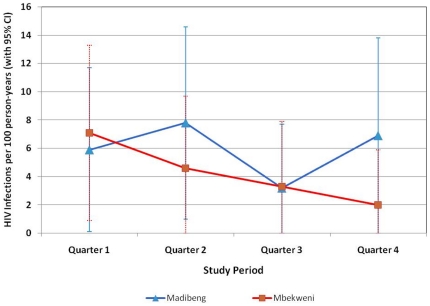
HIV incidence in the prospective cohort studies. Women enrolled in the 12-month cohort studies visited the CRC at 3, 6, 9, and 12 months after enrollment for HIV testing according to the algorithm presented in Figure 1. HIV incidence rates were calculated based on a Poisson distribution with PY at risk in the denominator. They are expressed as number of cases per 100 PY, with 95% confidence intervals (CI). HIV infection was assumed to have occurred at the mid-point between the last available negative test and first positive test.

HIV incidence rates based on seroconversions in the cohort studies are shown in Figure 2. Overall incidence rates for the 12-month period were 6.0/100 PY (95% CI 3.0, 9.0) in Madibeng and 4.5/100 PY (95% CI 1.8, 7.1) in Mbekweni. Rates in Madibeng varied by study quarter, while rates in Mbekweni declined steadily, but number of events per quarter were small and confidence intervals wide (Figure 2). Positive predictors of seroconversion in Madibeng were ever having had anal sex (HR 8.5, 95% CI 1.9, 37.9); self-assessment of HIV risk as moderate/high versus none/low (HR 3.2, 95% CI 1.1, 9.1); and having two or more male sexual partners versus one in the 3-month period prior to screening (HR 7.4, 95% CI 2.7, 20.6). In Mbekweni, having a current sexual partner who is HIV-positive (HR 3.8, 95% CI 1.1, 13.0) was positively associated with HIV seroconversion.

HIV incidence rates estimated by cross-sectional BED testing were 7.1/100 PY (95% CI 2.8, 11.3) in Madibeng and 5.8/100 PY (95% CI 2.0, 9.6) in Mbekweni.

### Pregnancy rates

**Figure 3:**
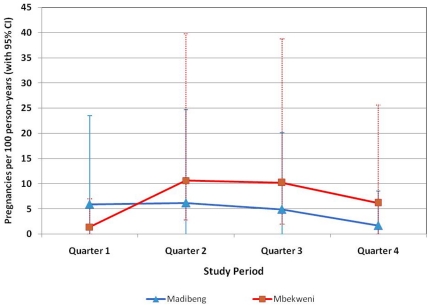
Pregnancy rates in the prospective cohort studies. Urine pregnancy tests were done at each study visit (screening, enrollment, and 3, 6, 9, and 12 months after enrollment in the cohort study). If test result was positive, the participant was to continue on study for follow-up per protocol. Estimated date of conception and estimated due date were to be recorded. If possible, follow-up was to continue for pregnancy outcome. Contraceptive counseling was provided and condoms were dispensed at each study visit.

In the cross-sectional studies, pregnancy prevalence was 2.5% (95% CI 1.4, 3.6) in Madibeng and 2.3% (95% CI 1.2, 3.3) in Mbekweni. In the cohort studies, overall pregnancy rates for the 12-month period were higher in Mbekweni (7.0/100 PY [95% CI 3.7, 10.3]) than Madibeng (4.8/100 PY [95% CI 2.2, 7.5]). In both districts, pregnancy rates decreased during the observation period (Figure 3). In Madibeng, decreased likelihood of condom use at last sex from baseline to follow-up was associated with incident pregnancy (6.0 [95% CI 1.6, 22.7], p<0.01). In Mbekweni, ever having cleansed the vagina before sex (reported at baseline) (7.7 [95% CI 1.7, 34.6], p<0.01); ever having cleansed the vagina after sex (reported at baseline) (12.6 [95% CI 2.7, 58.7], p<0.01); and not having used a condom in the last 7 days (reported during follow-up) (6.2 [95% CI 1.3, 29.6], p = 0.02) were predictors of incident pregnancy.

### Genital symptom rates

In the cross-sectional studies, the prevalence of self-reported genital symptoms (including genital discharge, lower abdominal pain, vaginal pruritus, dysuria, genital odor, genital sores/ulcers, swelling in groin area, and others) was 32.7% (95% CI 29.5, 36.0) in Madibeng and 9.9% (95% CI 7.8, 12.0) in Mbekweni. In the cohort studies, the incidence of self-reported genital symptoms during the 12-month period was 46.8/100 PY (95% CI 38.5, 55.2) in Madibeng and 21.5/100 PY (95% CI 15.8, 27.3) in Mbekweni. Over half of participants who reported genital symptoms during follow-up, 87 of 121 (72%) in Madibeng and 30 of 54 (56%) in Mbekweni, also reported symptoms at enrollment. In both districts, the percentage of participants reporting genital symptoms at each visit decreased throughout the 12-month observation period to 6% at Madibeng and 3.7% at Mbekweni.

### Willingness to participate in a microbicide trial

Almost all participants in each district (99–100%) reported that they are willing to participate in a microbicide trial.

## Discussion

The HIV prevalence in our studies among sexually active adult women 18–35 years of age was estimated to be 22–24% in Madibeng and Mbekweni, which is higher than the 2009 UNAIDS estimates of 13.6% for 15–24 year-old and 17.8% for 15–49 year-old South African women [1]. We may have measured a higher HIV prevalence because our study populations were (semi-)urban or because women who suspected that they were at risk for HIV were more interested in participating in our studies to access counseling, testing, and prevention services. On the other hand, women who already knew that they were HIV-positive were not eligible for study participation, which would suggest an underestimation of the true HIV prevalence in our studies.

In the cohort studies, HIV incidence based on seroconversions over the 12-month follow-up period was 6.0/100 PY in Madibeng and 4.5/100 PY in Mbekweni. BED-based HIV incidence estimates from the cross-sectional studies were slightly higher: 7.1 and 5.8/100 PY in Madibeng and Mbekweni, respectively. It should be noted, however, that the BED-based estimates were almost identical to the seroconversion rates in the first 6 months of the cohort studies (6.8 and 5.9/100 PY, respectively; data not shown). Data from the first 6 months of the cohort studies may be the most relevant for comparison with cross-sectional estimates because study participants may change their behavior in response to prevention messages and services received at the quarterly follow-up visits [12]. HIV seroconversion rates in Mbekweni indeed declined steadily during the 12 month follow-up period.

HIV incidence in both districts was high despite the fact that more than 85% of the women reported to only have had one sex partner in the past 3 months. This is most likely due to the high HIV prevalence in the communities: 12% of women in Madibeng and 37% of women in Mbekweni suspected that they had current sex partners that were HIV-positive. This proportion was most likely higher in Mbekweni than in Madibeng due to recent interventions in Mbekweni that promoted HIV testing. Furthermore, in both districts, condoms were not used consistently. In statistical models, determinants of both prevalent HIV infection at baseline and HIV seroconversion during the 12-month cohort studies were moderate or high perceived HIV risk, suspected positive or unknown serostatus of a current sexual partner, and ever having had anal sex. At entry into the cohort studies, women were asked to refrain from anal sex, and very few women reported anal sex throughout the studies (≤2% in both districts). However, anal sex is likely underreported in research studies due to social desirability bias [13]. Therefore, Phase III vaginal microbicide trial participants should be counselled on the increased HIV risk associated with anal sex, and the fact that vaginal microbicides are designed to protect women from vaginal acquisition of HIV only.

A few limitations of our data should be noted. The eligibility criteria for entry into our study limit generalizability of our results. The HIV prevalence rates in our paper apply to young, sexually active women who were not known to be HIV-infected or pregnant, and who agreed to be tested regularly for HIV. The total number of seroconversions in each prospective cohort study (15 and 11 in Madibeng and Mbekweni, respectively) were low and the 95% confidence intervals were therefore wide. Models of determinants of HIV prevalence and HIV seroconversion were adjusted for age only to maximize statistical power, and residual confounding may therefore have been present. The 95% confidence intervals of the cross-sectional BED-based HIV incidence estimates were also wide. Furthermore, we did not measure local false-recent rates or window periods and could therefore not adjust our BED estimates as recommended by WHO [14].

The use of a reliable contraceptive method was a study requirement because this would also be a requirement for enrollment into a vaginal microbicide trial of a microbicide containing a new chemical entity. Despite this, pregnancy incidence rates were high in both districts (4.8 and 7.0/100 PY in Madibeng and Mbekweni, respectively). The number of pregnancies decreased throughout the studies, perhaps due to contraceptive counseling and provision of condoms at each study visit. However, contraceptive services at the CRCs should be strengthened further to keep pregnancy rates as low as possible during future microbicide trials [15].

The prevalence and incidence of self-reported genital symptoms were high in Madibeng (33%, 46.8/100 PY) and Mbekweni (10%, 21.5/100 PY), but since laboratory testing for STIs and vaginal infections was not done in these studies, conclusions cannot be drawn about the prevalence and incidence of STIs or vaginal infections in these communities. Almost all participants (over 99%) in both Madibeng and Mbekweni expressed strong interest in future microbicide trials.

In conclusion, the Madibeng and Mbekweni populations might be suitable for Phase III microbicide trials provided that HIV incidence rates over time remain sufficiently high to support endpoint-driven trials. However, contraceptive services should be strengthened to keep pregnancy rates as low as possible.
